# Polyphenolic Compounds Analysis of Old and New Apple Cultivars and Contribution of Polyphenolic Profile to the In Vitro Antioxidant Capacity

**DOI:** 10.3390/antiox7010020

**Published:** 2018-01-24

**Authors:** Josephine Kschonsek, Theresa Wolfram, Annette Stöckl, Volker Böhm

**Affiliations:** Institute of Nutrition, Friedrich Schiller University Jena, Dornburger Straße 25-29, 07743 Jena, Germany; Josephine.Kschonsek@uni-jena.de (J.K.); resi.leipzig@googlemail.com (T.W.); st_annette@gmx.de (A.S.)

**Keywords:** apple polyphenols, HPLC-DAD, Folin-Ciocalteu, TEAC, ORAC, vitamin C, relative antioxidant activity

## Abstract

Polyphenols are antioxidant ingredients in apples and are related to human health because of their free radical scavenging activities. The polyphenolic profiles of old and new apple cultivars (*n* = 15) were analysed using high-performance liquid chromatography (HPLC) with diode array detection (DAD). The in vitro antioxidant capacity was determined by total phenolic content (TPC) assay, hydrophilic trolox equivalent antioxidant capacity (H-TEAC) assay and hydrophilic oxygen radical absorbance (H-ORAC) assay. Twenty polyphenolic compounds were identified in all investigated apples by HPLC analysis. Quercetin glycosides (203 ± 108 mg/100 g) were the main polyphenols in the peel and phenolic acids (10 ± 5 mg/100 g) in the flesh. The calculated relative contribution of single compounds indicated flavonols (peel) and vitamin C (flesh) as the major contributors to the antioxidant capacity, in all cultivars investigated. The polyphenolic content (HPLC data) of the flesh differed significantly between old (29 ± 7 mg/100 g) and new (13 ± 4 mg/100 g) cultivars, and the antioxidant capacity of old apple cultivars was up to 30% stronger compared to new ones.

## 1. Introduction

Apples (*Malus domestica*) have a growing scientific interest because many investigations have demonstrated their beneficial effects on human health. Through a variety of antioxidant ingredients, apples have been associated with reduced risks of degenerative and cardiovascular diseases, which are considered to be caused by oxidative stress, especially by free radicals and reactive oxygen species (ROS) [1]. Antioxidants have become even more a focus of research due to increased exposure to ROS.

Polyphenols are the most abundant antioxidants in the human diet and the major part of antioxidants in apples, rather than essential nutrients such as vitamin C [2,3]. Apples are one of the most important fruit sources of dietary polyphenolic compounds in the Western diet, due to the fact that they are consumed widely and are available throughout the year [4]. In Germany, they are the most popular type of fruit with an annual consumption of 25.9 kg per person [5]. Polyphenols represent a group of secondary metabolites with aromatic ring(s) bearing one or more hydroxyl moieties [6]. The large number of conjugated double bonds and hydroxyl groups is responsible for their antioxidant activity (AOA) [3,7]. There are five major groups of polyphenolic compounds found in apples: flavanols (catechin, epicatechin and procyanidins), phenolic acids (mainly chlorogenic acid), dihydrochalcones (phloretin glycosides), flavonols (quercetin glycosides) and anthocyanins (cyanidin) [8,9]. Various reports indicate that the polyphenolic profile and content as well as the antioxidant capacity (AOC) in apples are affected by different variables, such as cultivar, tissue zones, harvest time, geographic location and storage conditions [10,11,12].

In recent decades, new apple cultivars, such as Braeburn, Elstar, Golden Delicious, Granny Smith and Jonagold have become more popular among consumers in Germany and Western Europe, resulting in a gradual decrease of the cultivation of old cultivars. New cultivars are said to have a lower content of polyphenols. The contents of polyphenolic compounds of new cultivars were reduced by breeding due to the astringent taste and rapid enzymatic browning. Therefore, we hypothesize that the content of polyphenols and consequently the AOC of old apple cultivars are higher compared to new cultivars. The comparison of the polyphenol content as well as the occurrence and distribution of the main polyphenolic classes is barely reported for old and new apple cultivars. There is just one investigation that distinguished between old and new apple cultivars, but without separately analysing peel and flesh [13]. Apples provide a mixture of bioactive compounds, but the total phenolic or total flavonoid content often does not directly reflect the AOC [7]. There are some studies that have investigated the polyphenolic content of apple cultivars, using spectrophotometric assays or HPLC analysis. However, there is limited information about the relative contribution of each polyphenolic compound to the AOC because the number of standards used is restricted, especially with regard to the procyanidins and quercetin glycosides. In the present study, the contents of polyphenolic compounds in peel and flesh of different old and new apple cultivars were determined by using HPLC-DAD. Additionally, the in vitro AOC was measured using total phenolic content (TPC) assay, hydrophilic trolox equivalent antioxidant capacity (H-TEAC) assay and hydrophilic oxygen radical absorbance capacity (H-ORAC) assay. AOA data of 20 polyphenols and of vitamin C were determined and used to calculate the contribution of these antioxidants to the AOC.

## 2. Materials and Methods

### 2.1. Chemicals

Methanol and ethanol were from VWR (Darmstadt, Germany) and were like all other solvents used of HPLC grade. Trolox, 2,2′-azinobis-(3-ethylbenzothiazoline-6-sulphonic acid) diammonium salt (ABTS), Folin-Ciocalteu reagent, quercetin, (−)-epicatechin, procyanidin B1, procyanidin B2, procyanidin C1, p-coumaric acid, trans-cinnamic acid and phloridzin dihydrate were from Sigma–Aldrich (Taufkirchen, Germany). Gallic acid, (+)-catechin, chlorogenic acid, ferulic acid, coffeic acid, rutin trihydrate and acetic acid were purchased from Carl Roth (Karlsruhe, Germany). Avicularin was used from Phytolab (Vestenbergsgreuth, Germany). Protocatechuic acid, hyperoside, isoquercitrin, quercitrin and procyanidin A2 were from Extrasynthese (Genay, France). Reynoutrin was purchased from Carbosynth Limited (Berkshire, UK). 2,2′-Azobis(2-amidinopropane) dihydrochloride (AAPH) was from Fisher Scientific (Nidderau, Germany). HPLC grade water (18 MΩ) was prepared using a MicroPure purification system (Thermo Electron LED GmbH, Niederelbert, Germany). Sodium hydroxide, hydrochloric acid (Merck, Darmstadt, Germany), meta-phosphoric acid (Sigma–Aldrich) and all other chemicals were of analytical grade.

### 2.2. Sample Details and Preparation

Fifteen different apple cultivars were chosen for the investigation of polyphenolic compounds, vitamin C and AOC, including ten old apple cultivars (Berlepsch, Cox Orange, Dülmener Rosenapfel, Goldparmäne, Gravensteiner, James Grieve, Jonathan, Oldenburger, Ontario and Roter Boskoop) and five new apple cultivars (Braeburn, Elstar, Golden Delicious, Granny Smith and Jonagold). The new cultivars were picked at maturity, during harvest season in 2015, from a local market in Jena, Germany, and the old cultivars were from a private garden (Bad Blankenburg, Germany: 50° N–11° E). A pomologist authenticated the apple cultivars. One tree from each old cultivar was used for the gathering of fruits for the study. In total, three to five kg were harvested, depending on the size of the tree. The apples were picked from all sides of the tree. Fruit samples were peeled (2–3 mm thickness) and cored. The peel and flesh samples were homogenised separately, using a knife mill Grindomix GM 200 (Retsch, Haan, Germany), and stored at −25 °C until use. For all analyses (polyphenolic compounds, vitamin C and AOC), the peel and flesh samples were lyophilised.

### 2.3. Food Extraction

#### 2.3.1. Polyphenolic Compounds Analysis

Each apple cultivar was weighed into a 50 mL Falcon tube. For the determination of content of polyphenols, freeze-dried samples of apple peel (0.5 ± 0.05 g) and apple flesh (2.0 ± 0.05 g) were used. Samples were hydrolysed, using a method slightly modified from [14], involving the stepwise addition of hydrochloric acid (1 M), followed by addition of sodium hydroxide solution in 75% methanol (2.0 M) and meta-phosphoric acid (0.75 M). A volume of 2 mL (apple peel) or 4 mL (apple flesh), respectively, was used of each solvent. All steps were accompanied by shaking (30 s) and heating in a water bath (30 min, 37 °C). After hydrolysis, the samples were centrifuged (5 min, 3000× *g*) (5702 R, Eppendorf, Hamburg, Germany). The supernatants were collected in 250 mL round-bottomed flasks. The remaining residues were extracted with 20 mL methanol/water (70/30, *v*/*v*), using an ultrasonic bath (20 min, ≤40 °C). After centrifugation (5 min, 3000× *g*), the supernatants were collected. The extraction was repeated twice, while the time of ultrasonic treatment was changed to 10 min (in both extractions) and the volume of methanol/water to 10 mL (only in the last extraction). The extraction procedure was performed according to a modified method from [15]. The supernatants of hydrolysis and the combined extracts were mixed and rotary-evaporated, under reduced pressure, at 35 °C, to a small volume. The dried residues were dissolved in methanol/water (70/30, *v*/*v*, 5 mL). After centrifugation (5 min, 19,000× *g*), the samples were used for HPLC analysis.

#### 2.3.2. Antioxidant Capacity (AOC) Assays

Each apple cultivar was weighed into a 15 mL Falcon tube. For the AOC assays freeze-dried samples of apple peel (0.1 ± 0.01 g) and apple flesh (0.4 ± 0.05 g) were used. Samples were hydrolysed, as described in the previous Section 2.3.1, except that the volume of each solvent was decreased five-fold. After hydrolysis, the samples were centrifuged (5 min, 3000× *g*) (5702 R, Eppendorf, Hamburg, Germany) and the supernatants were collected in 10 mL volumetric flasks. The residues were extracted with 2 mL methanol/water (70/30, *v*/*v*) by shaking for 30 min. After centrifugation (5 min, 3000× *g*), the supernatants were collected and the extraction was repeated twice. The supernatants from hydrolysis and the combined extracts were mixed and used for AOC assays.

### 2.4. HPLC Analysis of Polyphenols

The polyphenolic composition was analysed using a diode array detector (L-7450A, Merck Hitachi, Darmstadt, Germany) according to the method from [16]. The column was a reversed-phase Luna C18 column (250 mm × 4.6 mm, particle size 5 μm, Phenomenex, Aschaffenburg, Germany) and was heated to 30 °C. An injection volume of 50 µL was used. The binary mobile phase consisted of 0.5% (*v*/*v*) acetic acid in water (solvent A) and methanol (solvent B), pumped at a flow rate of 0.8 mL/min, for a total run time of 160 min. Elution was performed using a gradient program: 0–2 min, 0% B isocratic; 2–6 min, linear gradient from 0% to 15% B; 6–12 min, 15% B isocratic; 12–17 min, linear gradient from 15% to 20% B; 17–35 min, 20% B isocratic; 35–90 min, linear gradient from 20% to 35% B; 90–132 min, 35% B isocratic, 132–150 min, linear gradient from 35% to 80% B, 150–160 min linear gradient from 80% to 0% B. The detector was set at 254, 280 and 320 nm for simultaneous monitoring of the different groups of polyphenols. The identification was performed by comparing retention times and DAD absorbance spectra with external standards (Table 1). Polyphenolic compound contents were quantified with a 7-point calibration curve of external standards. Linearity was given over the entire 7-point calibration curve. The limits of detection (LOD) and limits of quantification (LOQ) of the polyphenols were determined using the baseline noise signals in chromatograms of five solvent injections (Table S1).

### 2.5. Antioxidant Capacity (AOC)

The AOC assays were performed by using clear 96-well microplates (Kisker Biotech, Steinfurt, Germany) and a microplate reader FluoStar Optima (BMG Labtech, Offenburg, Germany), in accordance with [17].

#### 2.5.1. Total Phenolic Content (TPC) Assay

The TPC was evaluated by using the Folin–Ciocalteu method [18]. Thirty microliters of methanol/water (70/30, *v*/*v*) extract were mixed with 150 µL 1:10 diluted Folin–Ciocalteu reagent and 120 µL sodium carbonate solution (75 g/L) in wells of a 96-well microplate. After 2 h in darkness, at room temperature, the absorbance was measured at 740 nm in the microplate reader, at 30 °C. Gallic acid monohydrate (8.51–170.12 mg/L) was used as the standard for calibration and construction of a linear regression line and water acted as the blank. The TPC is expressed as gallic acid equivalents (GAE) in mg/100 g.

#### 2.5.2. Hydrophilic Trolox Equivalent Antioxidant Capacity (H-TEAC) Assay

The H-TEAC assay was performed using ABTS radical cation (ABTS^•+^) [19]. This stable radical cation was formed by mixing 10 mL ABTS solution (7 mM) with 10 mL potassium peroxodisulfate solution (2.45 mM). After 24 h at room temperature (darkness), the ABTS^•+^ stock solution was ready to use. The ABTS^•+^ working solution was prepared freshly each day by diluting the ABTS^•+^ stock solution with phosphate buffer (75 mM, pH 7.4) to an absorbance of 0.70 ± 0.05 at 730 nm. Twenty microliters of methanol/water (70/30, *v*/*v*) extract were mixed with 200 mL ABTS^•+^ working solution in a 96-well microplate. The decrease in absorbance at 730 nm, at 30 °C, was measured photometrically. Trolox solutions (12.5–250 µM) were used for constructing a regression line and water acted as blank. The AOC is expressed as trolox equivalents (TE) in mmol/100 g.

#### 2.5.3. Hydrophilic Oxygen Radical Absorbance Capacity (H-ORAC) Assay

The H-ORAC assay was evaluated using fluorescein and AAPH [20]. The fluorescein working solution (1.2 µM) was prepared freshly each day by diluting the fluorescein stock solution (0.12 mM, stored in fridge) 1:100 with phosphate buffer (75 mM, pH 7.4). Ten microliters of methanol/water (70/30, *v*/*v*) extract were mixed with 25 µL fluorescein working solution and 100 µL phosphate buffer, in microplate wells. Afterwards, the 96-well plate was pre-heated for 10 min at 37 °C. Following the addition of 150 µL of freshly prepared and ice-cooled AAPH solution (129 mM) in phosphate buffer, the reaction started, and the fluorescence intensity was measured each minute, for 4 h, at 37 °C. Fluorescence filters of 490 nm (excitation) and 520 nm (emission) were used in the microplate reader. Water acted as a blank and trolox standard solutions (0.1–2.0 mM) were used for calibration. Additionally, a negative control (water) was used for controlling the photostability of fluorescein. Therefore, 150 µL of phosphate buffer was utilised instead of AAPH solution. The calculation of the H-ORAC assay can be seen in the publication by [17]. The AOC is expressed as trolox equivalents (TE) in mmol/100 g.

### 2.6. Vitamin C Analysis

The vitamin C analysis was performed in accordance with [21]. Each apple cultivar was weighed into a screwed glass tube. Freeze-dried samples of apple peel (0.1 ± 0.01 g) and apple flesh (0.4 ± 0.05 g) were used. Samples were extracted three times with 5 mL meta-phosphoric acid (0.46 M) by shaking for 1 min. After each extraction, the samples were centrifuged (5 min, 3000× *g*). The supernatants were collected in 20 mL volumetric flasks. Two hundred microliters of each sample, 200 µL of calibration solution (5.7–567.8 µM, respectively) and 200 µL of distilled water (blank) were mixed with 300 µL trichloroacetic acid (0.31 M) and centrifuged (5 min, 17,000× *g*). Three hundred microliters of the supernatants were mixed with 100 µL DNP reagent (one volume of thiourea solution (0.83 M in distilled water), one volume of copper sulphate solution (24 mM in distilled water) and 20 volumes of 2,4-dinitrophenylhydrazine solution (0.11 M in 4.5 M sulfuric acid). The mixture was heated in a thermomixer (1 h, 60 °C). The samples were cooled in an ice bath for 5 min. Afterwards, 400 µL sulfuric acid (8.56 M) was added and mixed. The samples were placed into darkness for 20 min. Finally, the samples were decanted into semi-micro cuvettes and measured using a photometer at 520 nm.

### 2.7. Statistical Analysis

All analyses were done in triplicate. The data are expressed as mean ± standard deviation (SD) and were analysed using SPSS procedures (version 22.0, Statistical Package for the Social Sciences, Chicago, IL, USA). *p*-Values < 0.05 were considered significant. The homogeneity of variances for all data was assumed by Levene’s test. The one factorial analysis of variance (ANOVA) was used followed by the Student-Newmann-Keuls (S-N-K) procedure for assessing differences between all 15 apple cultivars. The unpaired *t*-test was used to statistically compare the average out of all ten old cultivars and the average out of all five new cultivars. Correlations were tested by using the Pearson procedure, in which the *p*-value was considered to be significant at *p* < 0.01.

## 3. Results and Discussion

### 3.1. Quantification of Polyphenolic Compounds and Vitamin C

In the apples investigated, 20 polyphenolic compounds of four sub-classes (flavanols, phenolic acids, dihydrochalcones and flavonols) were identified by using retention times and absorption maxima of reference compounds (Table 1). The types of polyphenolic compounds detected in the apple cultivars were similar to previous studies, including the closely related quercetin glycosides that are often difficult to separate [13,15].

As shown in Table 2 and Table 3, the content of polyphenolic compounds varied greatly between apple peel and flesh. The total polyphenols determined by HPLC ranged from 99.6 ± 5.4 to 495.3 ± 44.0 mg/100 g in the peel, whereas the flesh had a 3.0 to 28.4 times lower content (9.6 ± 0.6 to 41.6 ± 2.3 mg/100 g). These results are comparable with other investigations [9,11,22,23].

The apple cultivars Ontario and Jonathan had the highest total polyphenol content in the peel and the cultivars Roter Boskoop and Dülmener Rosenapfel in the flesh. The cultivar Golden Delicious contained the significantly lowest content of total polyphenols in both peel and flesh, being in line with studies previously reported [12,24]. Furthermore, the occurrence and distribution of the main polyphenol classes differed between peel and flesh. Flavonols were the predominant group (72.2%) in the peel, followed by flavanols (14.7%), phenolic acids (8.3%) and the dihydrochalcone phloridzin (4.7%). The flavonols, which included quercetin and its glycosides, ranged from 52.0 ± 4.3 (Gravensteiner) to 430.0 ± 60.9 mg/100 g (Ontario). A high content of quercetin was found in the peel, due to hydrolysis with 1 M HCl, which efficiently released quercetin from sugar components [25,26]. The concentrations of the quercetin glycosides generally followed the order: hyperoside > quercitrin > avicularin > reynoutrin > isoquercitrin > rutin, with interchanging of avicularin and quercitrin depending on the apple cultivar, and is almost coincident with results of other studies [27,28,29]. The quercetin glycoside hyperoside was the major polyphenolic compound (30.3% of total polyphenols content) in the peel of all apple cultivars investigated and can be regarded as a marker for apple peel. Looking at the polyphenol profile in the flesh, the phenolic acids (43.0%) and the flavanols (41.1%) were the predominant groups, followed by the dihydrochalcon phloridzin (14.0%). The flavonols were missing in the flesh. The apple peel and flesh differed distinctly in the phenolic glycoside composition (Table 2 and Table 3). The flesh contained the phloretin glycoside phloridzin, whereas peel possessed both phloridzin and quercetin glycosides [25]. Within the phenolic acids, chlorogenic acid was the major compound in the peel (6.3 ± 3.3 10 mg/100 g) and in the flesh (5.0 ± 3.0 mg/100 g).

As described by Guyot et al., (1998), the phenolic acid content decreased from the peel to the flesh [10]. A total of six flavanols could be quantified in both peel (36.3 ± 11.7 mg/100 g) and flesh (9.6 ± 5.0 mg/100 g), but their concentrations in the flesh were much lower than in the peel. In addition to (+)-catechin and (−)-epicatechin, the procyanidin isomers B1, B2, C1 and A2 were identified, even if they were not detectable in all apple cultivars. In previous studies, the flavanols, especially the procyanidins, were the major class of apple polyphenols in both flesh and peel, representing more than 80% of the total polyphenol content (HPLC data) [8,11,13,23,28]. However, the content and percentage of flavanols obtained in this study were lower than the quantities reported in other research. One possible reason for this may be the crushing of the apples without adding an antioxidant, like ascorbic acid. Flavanol monomers and procyanidins are good substrates for polyphenol oxidase and are directly involved in enzymatic oxidation, occurring when apples are crushed [10].

In addition to the determination of polyphenolic compounds, the vitamin C content of the apples was analysed. The vitamin C content in the peel ranged from 99.2 ± 10.0 (Granny Smith) to 300.9 ± 10.8 (Ontario) mg/100 g, and in the flesh from 50.4 ± 2.4 (Golden Delicious) to 143.4 ± 1.8 (Ontario) mg/100 g. The vitamin C results are similar to other investigations [13,30,31], but tended to be higher, which may be due to our method. In the spectrophotometric method with DNP reagent, used in this research, the total content of vitamin C was analysed, i.e., the content of ascorbic acid and dehydroascorbic acid present in the apples. The vitamin C concentration significantly correlated with the polyphenol content, in both flesh and peel (*r* = 0.515; *p* < 0.001 and *r* = 0.736; *p* < 0.001, respectively). Therefore, as with the polyphenols, the content of vitamin C in the peel was higher (1.5- to 3.3-fold greater), compared to the flesh. A possible reason for the higher content of polyphenols and vitamin C in the peel, compared to the flesh, might be the barrier function of the peel against external biotic and abiotic stress, to which apples are often exposed [32]. Thus, a higher concentration of antioxidants appears to be useful in the peel.

### 3.2. Antioxidant Capacity (AOC)

As shown in Table 2 and Table 3, peel extracts had a stronger AOC than the flesh extracts, being comparable to studies previously reported [3,4,27,33,34]. The apple cultivar Oldenburger possessed the significantly highest AOC in the peel, and Jonathan in the flesh. The cultivars Golden Delicious and Braeburn had the significantly lowest AOC measured by all methods, in both peel and flesh. The total polyphenol content (HPLC data) significantly correlated with the values of the AOC results (TPC: 0.891; H-TEAC: 0.886; H-ORAC: 0.908; *p* < 0.001). The significant correlation between the vitamin C content and the AOC results (TPC: 0.867; H-TEAC: 0.861; H-ORAC: 0.883; *p* < 0.001) was lower compared to the polyphenols and suggest that the polyphenols have a more significant contribution to the AOC. The best correlation was found between the total polyphenols (HPLC data) and the H-ORAC results (*r* = 0.908; *p* < 0.001). The reaction mechanism of the ORAC assay is based on free peroxyl radicals, which are commonly found in the human body, making the reaction biologically relevant [34]. The H-ORAC values of the peel extracts showed the best positive linear correlation with the total flavanols (*r* = 0.767; *p* < 0.001) in the decreasing order of (−)-epicatechin > procyanidin C1 > (+)-catechin > procyanidin A2. Next to the flavanols, the total flavonols significantly correlated with the H-ORAC results (*r* = 0.651; *p* < 0.001), but the flavanols had an even better linear correlation. Similar observations for the flavanols were found in the flesh extracts (*r* = 0.784; *p* < 0.001), with some differences in the order: (+)-catechin > (−)-epicatechin > procyanidin A2 > procyanidin B2. These results show that the flavanols are major contributors to the AOC (H-ORAC), in both peel and flesh.

### 3.3. Relative Contribution to AOC

To indicate the relative contribution of single antioxidants to the AOC of each apple sample, the relative antioxidant activities (RAA) of the 20 polyphenolic compounds and vitamin C were calculated. The RAA resulted from the quotient of the molar AOA value of the H-ORAC assay of the corresponding polyphenol standard, and the molar concentration at which the H-ORAC was obtained.

RAA = AOA/C (1)

The RAA of the polyphenols showed the following order: procyanidin B1 (10.4) and procyanidin C1 (10,4) > procyanidin B2 (9.6) > rutin trihydrate (9.1) > phloridzin dihydrate (8.7) > reynoutrin (8.6) > hyperoside (7.9) > isoquercitrin (7.2) > quercetin (6.8) > avicularin (6.7) > quercitrin (6.6) > procyanidin A2 (6.0) > (−)-epicatechin (5.0) > p-coumaric acid (4.9) and (+)-catechin (4.9) > chlorogenic acid (4.7) > protocatechuic acid (4.3) > caffeic acid (4.0) > ferulic acid (2.9) > gallic acid (1.7) > vitamin C (0.9).

The relative contribution of a single compound to the AOC of apple extracts was obtained as follows:

contribution [%] = C_HPLC_ × (RAA/AOC) × 100 (2)

C_HPLC_ is the molar concentration of an individual compound, determined by HPLC analysis, RAA is the RAA value of the corresponding standard and AOC is the H-ORAC value of the apple sample. The total relative contribution is a sum of all calculated relative contribution values of the polyphenols determined in each apple sample.

**Figure 1:**
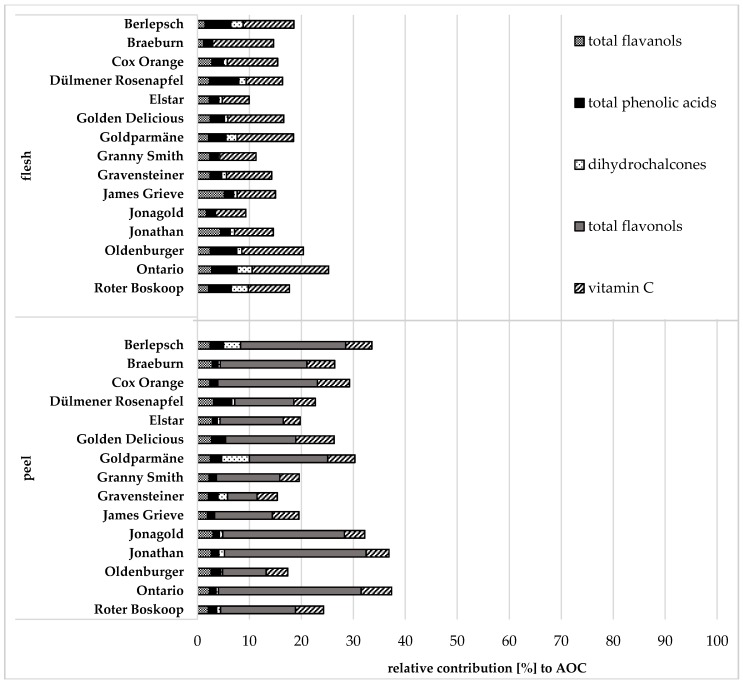
Relative contributions of the main polyphenol groups and vitamin C to the antioxidant capacity (AOC) of different apple cultivars in flesh and peel, calculated using H-ORAC results; H-ORAC: hydrophilic oxygen radical absorbance capacity.

Vitamin C has been considered to be one of the most prevalent antioxidant components in apples. However, as Lee et al. (2003) reported, dietary polyphenols have much stronger antioxidant activities than vitamin C [7], being confirmed by our investigations. A comparison of the measured AOC with the calculated H-ORAC value (sum of contributions of polyphenols) showed that only 26.1% (between 15.4% and 37.4%) of the AOC of the apple peel and 15.9% (between 9.3% and 25.3%) of the AOC of the apple flesh were due to the compounds analysed (Figure 1).

All results calculated were smaller than those of the values measured, suggesting unquantified polyphenolic compounds and possible synergisms and/or antagonisms among the polyphenols [3,7]. The values calculated were smaller than those reported by other authors, due to possibly different apple cultivars, AOC assays and methods for extraction and quantification of polyphenolic compounds [3,35,36].

Hyperoside had the highest contribution (6.8%) to the AOC in the peel, followed by quercitrin (2.1%) and avicularin (2.0%), whereas in the flesh, phloridzin and chlorogenic acid (1.2%) contributed the most, followed by caffeic acid (1.0%) and procyanidin A2 (0.8%). Considering the content of each polyphenolic compound, the total polyphenols had an average contribution of 21.2% (11.5–32.5%) to the AOC in the peel and of 6.8% (2.9–10.5%) to AOC in the flesh. In the apple peel, an average of 15.9% of the calculated AOC was due to the flavonols, 2.5% due to the flavanols, 1.7% due to the phenolic acids and 1.1% due to the dihydrochalcone phloridzin (Figure 1). Of the three polyphenolic groups identified in the flesh, the total phenolic acids provided 3.0%, followed by the total flavanols (2.6%) and the dihydrochalcone phloridzin (1.2%) (Figure 1). These results are in agreement with other publications, showing a strong AOA for flavanols and flavonols, being thus main contributors to the AOC of apples [3,7,8,36,37]. In addition to the polyphenols, vitamin C contributed to the AOC of peel (4.9%) and flesh (9.1%). These results clearly indicate that in the peel flavonols rather than vitamin C made the major contribution to AOC of apples, whereas in the flesh, vitamin C provided the highest contribution.

### 3.4. Comparison of Old and New Apple Cultivars

In the present study, the extracts of flesh and peel of old apple cultivars (*n* = 10) and new apple cultivars (*n* = 5) were analysed in terms of their polyphenolic profiles, vitamin C content and AOC. The flesh of old cultivars had significantly higher content of polyphenolic compounds (HPLC data) and vitamin C, compared to the flesh of new cultivars, which led also to a higher AOC in TPC, H-TEAC and H-ORAC assays for the old ones. In contrast to the flesh, the polyphenolic content and the AOC of the apple peel did not differ between old and new cultivars, whereas the vitamin C content was significantly higher in the peel of old cultivars. As shown in Table 4, the polyphenolic profile of the new apple cultivars was characterised by a lower content of total flavanols (flesh) and total phenolic acids (flesh and peel) and a lower content of phloridzin (flesh and peel), compared to the old ones. The lower content of flavanols and phloridzin resulted in a lower AOC in the new apple cultivars, because monomeric and oligomeric flavan-3-ols and phloridzin are strong antioxidants with high AOAs.

Phenolic acids and flavanols are responsible for an astringent taste and a rapid enzymatic browning of apples [38]. Since consumers prefer sweet-flavoured and low enzymatic browning apples, the content of phenolic acids and flavanols decreased by breeding new apple cultivars. New apple cultivars tend to be genetically impoverished because they are almost entirely due to six similar strain varieties, but old cultivars have a higher vitality [39]. Presumably, the flesh of old cultivars contains higher levels of polyphenolic compounds, vitamin C and AOC due to species diversity. Vrhovsek et al., (2004) also showed that old cultivars (whole fruit, without separation into peel and flesh) have a higher average content of total polyphenols and, in particular, of flavanols, compared to new cultivars [13]. A reason why there are no differences in the peel between old and new cultivars may be the protective function of antioxidants, like polyphenolic compounds, in the peel; thus, they are necessary ingredients to counter biotic and abiotic factors.

## 4. Conclusions

The results of this research clearly indicated that flavonols, rather than vitamin C, make the major contribution to AOC in apple peel, whereas in the flesh, vitamin C provides the highest contribution next to the flavanols. The flesh of old apple cultivars had a higher content of polyphenolic compounds and vitamin C, resulting in a higher AOC compared to new apple cultivars. For this reason, it is recommended to include preferably old apple cultivars, such as Jonathan, Ontario and Oldenburger, in the daily diet. Additionally, it is advisable to consume the apple with peel, due to its higher polyphenolic content and stronger AOC. Both aspects can help to increase the polyphenol intake and the AOC within the daily diet.

## Figures and Tables

**Table 1:** Characterization (retention time [t_R_], absorption maximum [λ_max_]) of 20 polyphenols of apples used as reference compounds in HPLC-DAD.

Group of Polyphenols	Phenolic Compound	Synonyms	t_R_ [min]	λ_max_ [nm]	λ [nm]
Flavanols	Procyanidin B1		21.09	281	280
	(+)-Catechin		25.71	281	280
	Procyanidin B2		29.04	281	280
	Procyanidin C1		39.87	275	280
	(−)-Epicatechin		43.11	280	280
	Procyanidin A2		68.85	280	280
Phenolic acids	Gallic acid	Chlorogenic acid	12.64	272	280
	Protocatechuic acid		18.80	261, 298	254
	5-*O*-Caffeoylquinic acid		31.12	326	320
	Caffeic acid		36.05	324	320
	p-Coumaric acid		57.10	310	320
	Ferulic acid		66.15	324	320
Dihydrochalcones	Phloretin-2-*O*-β-glucoside	Phloridzin *	106.84	287	280
Flavonols	Quercetin-3-*O*-galactoside	Hyperoside	94.19	259, 348	254
	Quercetin-3-*O*-glucoside	Isoquercitrin	96.96	259, 351	254
	Quercetin-3-*O*-rutinoside	Rutin *	97.68	259, 348	254
	Quercetin-3-*O*-xyloside	Reynoutrin	101.23	260, 348	254
	Quercetin-3-*O*-arabinoside	Avicularin	112.03	260, 347	254
	Quercetin-3-*O*-rhamnoside	Quercitrin	116.37	260, 347	254
	Quercetin		145.33	256, 372	254

* Phloridzin as Phloridzin dehydrate; Rutin as Rutin trihydrate; λ: wavelength used in HPLC-DAD.

**Table 2:** Average content of vitamin C, polyphenolic compounds and antioxidant capacity values in the peel of different apple cultivars.

Apple Cultivars	1	2	3	4	5	6	7	8	9	10	11	12	13	14	15
vitamin C	207.5	215.7	196.9	155.5	186.4	124.3	225.8	99.2	117.4	184.0	189.1	210.2	256.0	300.9	204.4
procyanidin B1	n.d.	n.d.	1.3	4.8	n.d.	n.d.	0.9	n.d.	1.8	1.0	n.d.	0.9	n.d.	1.3	n.d.
(+)-catechin	2.3	4.9	2.7	6.8	9.0	1.5	3.5	3.4	3.2	3.0	8.3	7.4	11.7	7.4	3.6
procyanidin B2	7.5	7.2	7.8	5.2	9.0	8.2	9.6	5.1	4.4	6.8	7.6	8.3	8.2	6.2	6.7
procyanidin C1	6.6	7.0	5.2	6.6	9.5	n.d	6.6	6.9	n.d.	5.3	7.7	7.6	8.3	8.4	5.9
(−)-epicatechin	13.2	11.4	3.0	8.9	18.5	1.1	7.9	3.1	2.2	4.2	16.4	13.2	17.8	11.3	6.4
procyanidin A2	4.1	10.5	7.7	7.3	10.4	5.1	10.2	2.6	12.4	12.1	9.8	5.1	8.7	5.7	5.0
total flavanols	33.7	41.0	27.7	39.5	56.4	15.8	38.7	21.0	23.9	32.3	49.8	42.4	54.7	40.4	27.5
SD	±4.2	±2.7	±2.7	±1.5	±4.1	±3.3	±3.6	±1.8	±4.4	±3.8	±3.7	±4.1	±3.6	±3.3	±1.3
gallic acid	4.3	3.9	3.8	4.9	3.8	4.0	7.2	3.5	4.8	4.7	4.2	4.8	4.3	3.9	3.7
protocatechuic acid	1.0	1.3	1.5	2.3	1.9	0.6	1.9	0.8	1.1	1.3	2.0	3.8	1.9	2.4	2.5
chlorogenic acid	18.1	4.9	4.8	7.0	5.3	5.9	5.1	5.2	3.9	4.0	6.7	6.1	7.1	5.5	5.2
caffeic acid	6.6	1.1	0.4	11.1	1.2	0.6	6.2	0.1	3.2	0.8	1.2	1.4	10.4	3.5	2.2
p-coumaric acid	2.6	1.8	1.7	5.9	2.3	1.4	3.5	1.4	2.4	1.9	2.0	2.3	4.1	1.9	2.2
ferulic acid	1.1	0.4	1.0	2.3	0.7	1.3	2.3	0.7	0.7	0.7	1.4	1.4	1.4	1.0	1.4
total phenolic acids	33.6	13.5	13.2	33.6	15.2	13.7	26.2	11.7	16.1	13.4	17.5	19.7	29.3	18.1	17.1
SD	±6.5	±1.8	±1.7	±3.3	±1.7	±2.2	±2.1	±2.0	±1.6	±1.7	±2.1	±1.9	±3.4	±1.6	±1.4
phloridzin	37.1	4.6	1.5	6.1	8.3	1.5	63.8	0.8	15.3	6.1	8.8	14.5	6.8	6.9	8.0
hyperoside	94.7	103.5	91.2	44.7	102.8	18.31	83.4	28.6	8.0	80.3	168.1	190.9	60.2	189.4	79.3
isoquercitrin	30.9	19.1	8.1	12.6	18.5	4.87	44.8	11.0	5.6	9.7	29.6	18.9	13.8	24.5	9.8
Rutin	24.3	6.6	1.4	14.5	7.2	1.16	1.9	2.4	1.9	0.9	9.8	1.9	1.9	15.5	6.8
reynoutrin	18.6	19.6	15.6	10.6	19.3	6.99	15.7	11.2	4.1	10.2	27.9	39.8	20.1	40.0	15.4
avicularin	35.0	19.6	30.3	18.1	28.4	12.79	23.7	13.3	9.7	22.7	33.1	62.4	29.5	51.4	23.8
quercitrin	36.2	19.7	18.4	25.5	17.9	15.94	15.4	23.4	15.4	24.0	50.2	40.6	17.1	84.1	20.9
quercetin	17.7	13.5	16.9	7.6	13.8	8.54	12.5	8.8	7.3	14.0	22.4	32.5	12.0	25.1	9.5
total flavonols	257.3	201.6	181.8	133.4	207.9	68.6	197.4	98.6	52.0	161.8	340.9	386.9	154.4	430.0	165.5
SD	±26.6	±33.3	±30.1	±12.7	±32.8	±6.2	±27.7	±8.9	±4.3	±26.4	±54.0	±62.7	±18.8	±60.9	±25.3
total polyphenols	361.7	260.8	224.2	212.5	287.8	99.6	326.1	132.1	107.3	213.5	416.9	463.5	245.2	495.3	218.1
SD	±22.2	±22.7	±20.4	±9.7	±22.5	±5.4	±22.2	±7.8	±4.6	±17.7	±37.8	±42.9	±13.5	±44.0	±17.5
TPC	889.6	926.0	687.6	889.5	1320.4	521.9	1238.2	581.0	638.8	772.5	1224.2	1212.8	1590.5	1078.7	960.6
TEAC	5.3	6.0	4.4	5.9	8.5	2.4	7.7	3.4	3.4	5.0	9.1	9.9	12.8	8.3	6.1
ORAC	21.1	20.6	16.3	19.2	29.0	8.6	22.0	13.6	15.3	18.3	24.6	24.4	31.4	26.2	19.3

1. Berlepsch; 2: Braeburn; 3: Cox Orange; 4: Dülmener Rosenapfel; 5: Elstar; 6: Golden Delicious; 7: Goldparmäne; 8: Granny Smith; 9: Gravensteiner; 10: James Grieve; 11: Jonagold; 12: Jonathan; 13: Oldenburger; 14: Ontario; 15: Roter Boskoop; SD: standard derivation; n.d.: not detected; TPC: total phenolic content; TEAC: trolox equivalent antioxidant capacity; ORAC: oxygen radical antioxidant capacity; single compounds and total concentrations in mg/100 g freeze dried material; TPC in mg GAE/100 g freeze dried material; TEAC and ORAC in mmol TE/100 g freeze dried material; GAE: gallic acid equivalents; TE: trolox equivalents.

**Table 3:** Average content of vitamin C, polyphenolic compounds and antioxidant capacity values in the flesh of different apple cultivars.

Apple Cultivars	1	2	3	4	5	6	7	8	9	10	11	12	13	14	15
vitamin C	108.0	112.3	99.5	83.2	67.7	50.4	94.7	54.7	77.0	85.5	57.4	118.2	124.8	134.4	106.8
procyanidin B1	0.5	0.5	n.d.	6.8	n.d.	0.2	0.3	n.d.	0.8	0.7	n.d.	4.0	0.4	n.d.	n.d.
(+)-catechin	0.8	0.5	1.4	1.6	2.5	0.3	0.8	1.8	0.3	1.1	0.5	4.9	1.0	1.5	1.3
procyanidin B2	0.9	1.3	1.9	2.5	1.8	1.4	1.7	1.4	1.4	1.6	2.0	2.7	1.9	2.3	2.0
procyanidin C1	1.3	n.d.	n.d.	n.d.	n.d.	n.d.	n.d.	n.d.	n.d.	1.3	n.d.	1.7	n.d.	n.d.	n.d.
(−)-epicatechin	0.3	0.5	1.2	2.2	2.0	0.3	1.4	1.5	0.8	1.4	0.7	4.7	1.5	2.2	1.5
procyanidin A2	2.0	1.4	6.6	3.5	4.5	2.1	2.4	1.6	5.3	11.0	3.1	5.9	5.0	2.6	4.0
total flavanols	5.9	4.2	11.1	16.6	10.8	4.4	6.6	6.3	8.6	17.1	6.3	23.8	9.6	8.6	8.8
SD	±0.6	±0.5	±2.6	±2.1	±1.2	±0.9	±0.8	±0.2	±2.0	±4.0	±1.2	±1.5	±1.8	±0.5	±1.2
gallic acid	1.7	1.6	1.7	1.6	1.6	1.9	2.0	1.4	1.7	2.2	1.7	1.9	1.7	1.6	1.7
protocatechuic acid	0.2	0.1	0.	0.1	0.1	0.1	0.1	0.1	0.1	0.1	0.1	0.1	0.2	0.1	0.3
chlorogenic acid	8.4	2.0	3.2	10.8	3.6	1.4	5.8	1.7	3.3	3.3	2.1	4.4	8.3	7.6	8.6
caffeic acid	5.6	0.6	1.5	6.8	1.7	0.5	1.0	0.5	0.6	0.7	0.4	2.2	5.0	3.8	6.0
p-coumaric acid	0.7	0.4	0.6	0.8	0.6	0.4	0.7	0.4	0.7	0.6	0.5	0.7	0.9	0.7	0.8
ferulic acid	0.2	0.1	0.1	0.2	0.1	0.1	0.3	0.1	0.1	0.2	0.2	0.1	0.4	0.1	0.2
total phenolic acids	16.7	4.8	7.1	20.3	7.6	4.4	9.9	4.2	6.5	7.1	5.0	9.4	16.4	13.9	17.5
SD	±3.4	±0.8	±1.2	±4.4	±1.3	±0.7	±2.1	±0.7	±1.2	±1.3	±0.8	±1.6	±3.3	±2.9	±3.5
phloridzin	7.3	0.7	2.3	4.7	3.6	0.9	5.2	0.7	2.3	2.3	0.7	3.6	2.9	7.4	12.3
total polyphenols	29.8	9.6	20.5	41.6	20.8	9.6	21.7	11.2	17.4	26.5	12.0	36.8	28.9	29.9	38.6
SD	±2.8	±0.6	±1.8	±3.4	±1.4	±0.7	±2.9	±0.7	±1.5	±2.9	±1.0	±1.9	±2.5	±2.6	±3.9
TPC	220.7	143.6	219.8	300.3	276.4	136.5	252.0	163.3	174.9	246.3	177.5	361.7	242.9	217.6	334.1
TEAC	1.1	0.8	1.3	1.8	1.5	0.8	1.2	0.9	0.9	1.5	1.0	2.3	1.5	1.2	2.0
ORAC	5.7	4.9	5.2	6.1	6.7	2.4	4.5	4.0	4.5	5.9	5.0	8.1	5.4	4.7	6.9

1. Berlepsch; 2: Braeburn; 3: Cox Orange; 4: Dülmener Rosenapfel; 5: Elstar; 6: Golden Delicious; 7: Goldparmäne; 8: Granny Smith; 9: Gravensteiner; 10: James Grieve; 11: Jonagold; 12: Jonathan; 13: Oldenburger; 14: Ontario; 15: Roter Boskoop; SD: standard derivation; n.d.: not detected; TPC: total phenolic content; TEAC: trolox equivalent antioxidant capacity; ORAC: oxygen radical antioxidant capacity; single compounds and total concentrations in mg/100 g freeze dried material; TPC in mg GAE/100 g freeze dried material; TEAC and ORAC in mmol TE/100 g freeze dried material; GAE: gallic acid equivalents; TE: trolox equivalents.

**Table 4:** Comparison of content of vitamin C, phenolic compounds and antioxidant capacity results between old apple cultivars (*n* = 10) and new apple cultivars (*n* = 5).

	Apple Flesh			Apple Peel		
	Old Cultivars	New Cultivars	*p*	Old Cultivars	New Cultivars	*p*
ascorbic acid	102.6 ± 19.3	68.5 ± 23.7	**<0.001**	203.5 ± 50.5	162.9 ± 45.9	**0.012**
procyanidin B1	1.0 ± 1.9	1.0 ± 0.1	0.081	1.9 ± 1.6	n.d.	-
(+)-catechin	1.5 ± 1.2	1.1 ± 0.9	0.351	5.1 ± 3.0	5.4 ± 3.0	0.745
procyanidin B2	1.9 ± 0.5	1.6 ± 0.3	0.061	7.1 ± 1.5	7.4 ± 1.4	0.427
procyanidin C1	1.5 ± 0.2	n.d.	-	6.7 ± 1.2	7.8 ± 1.1	**0.016**
(−)-epicatechin	1.7 ± 1.2	1.0 ± 0.7	**0.034**	8.8 ± 4.9	10.1 ± 7.2	0.543
procyanidin A2	4.8 ± 2.6	2.5 ± 1.2	**<0.001**	7.8 ± 2.9	7.7 ± 3.4	0.855
total flavanols	11.2 ± 5.2	6.4 ± 2.5	**<0.001**	36.1 ± 8.8	36.8 ± 16.5	0.883
gallic acid	1.8 ± 0.2	1.6 ± 0.2	0.054	4.6 ± 1.0	3.9 ± 0.3	**0.001**
protocatechuic acid	0.1 ± 0.1	0.1 ± 0.0	**0.005**	2.0 ± 0.8	1.3 ± 0.6	**0.012**
chlorogenic acid	6.4 ± 2.7	2.1 ± 0.8	**<0.001**	6.7 ± 4.0	5.6 ± 0.7	0.159
caffeic acid	3.3 ± 2.4	0.8 ± 0.5	**<0.001**	4.6 ± 3.7	0.8 ± 0.5	**<0.001**
p-coumaric acid	0.7 ± 0.1	0.5 ± 0.1	**<0.001**	2.8 ± 1.3	1.8 ± 0.4	**<0.001**
ferulic acid	0.2 ± 0.1	0.1 ± 0.1	**0.003**	1.3 ± 0.6	0.9 ± 0.4	**0.007**
total phenolic acids	12.5 ± 4.9	5.2 ± 1.3	**<0.001**	22.0 ± 7.7	14.3 ± 2.1	**<0.001**
phloridzin	5.0 ± 3.1	1.1 ± 0.7	**<0.001**	16.6 ± 18.7	4.8 ± 3.5	**0.002**
hyperoside	n.d.	n.d.	-	92.2 ± 55.8	84.2 ± 57.1	0.657
isoquercitrin	n.d.	n.d.	-	17.8 ± 11.9	16.6 ± 8.6	0.716
rutin	n.d.	n.d.	-	7.1 ± 7.9	5.4 ± 3.3	0.319
reynoutrin	n.d.	n.d.	-	19.0 ± 11.5	17.0 ± 7.6	0.550
avicularin	n.d.	n.d.	-	30.6 ± 15.2	21.4 ± 8.4	**0.035**
quercitrin	n.d.	n.d.	-	29.8 ± 20.3	25.4 ± 13.1	0.455
quercetin	n.d.	n.d.	-	15.5 ± 7.9	13.4 ± 5.2	0.366
total flavonols	n.d.	n.d.	-	212.1 ± 112.3	183.5 ± 99.6	0.410
total polyphenols	28.7 ± 7.4	12.6 ± 4.4	**<0.001**	286.8 ± 118.8	239.4 ± 118.6	0.214
TPC	257.0 ± 56.7	179.5 ± 52.3	**<0.001**	995.9 ± 281.6	914.7 ± 331.3	0.163
TEAC	1.5 ± 0.4	1.0 ± 0.3	**<0.001**	6.9 ± 2.8	5.9 ± 2.7	0.051
ORAC	5.7 ± 1.3	4.6 ± 1.6	**<0.001**	21.4 ± 5.4	19.2 ± 7.8	0.060

*p*-Values represent results from unpaired *t*-test (*p* < 0.05; significant values are shown in bold); n.d.: not detected; TPC: total phenolic content; TEAC: trolox equivalent antioxidant capacity; ORAC: oxygen radical antioxidant capacity; single compounds and total concentrations in mg/100 g freeze dried material; TPC in mg GAE/100 g freeze dried material; TEAC and ORAC in mmol TE/100 g freeze dried material; GAE: gallic acid equivalents; TE: trolox equivalents.

## Supplementary Materials

The following are available online at http://www.mdpi.com/2076-3921/7/1/20/s1, Table S1: Calibration characteristics and limits of detection (LOD) and limits of quantification (LOQ) of reference compounds in HPLC-DAD.
