# Supplementary material for: Polyphenolic Compounds Analysis of Old and New Apple Cultivars and Contribution of Polyphenolic Profile to the In Vitro Antioxidant Capacity
Source: Antioxidants (Basel). 2018 Jan 24;7(1):20. doi: 10.3390/antiox7010020 (PMC5789330; doi:10.3390/antiox7010020)
Supplement: Supplementary file 1 [file antioxidants-07-00020-s001.pdf]

Table S1. Calibration characteristics and limits of detection (LOD) and limits of quantification (LOQ) of reference compounds in HPLC-DAD.

Phenolic Compound	c (μg/mL)	Calibration Curve Equation	Linearity *	LOD (μg/mL)	LOQ (μg/mL)
Procyanidin B1	0.5–20	y = 9.537x − 1.564	0.9982	0.5	2.8
(+)-Catechin	0.5–50	y = 16.083x − 7.072	0.9993	0.9	2.2
Procyanidin B2	0.5–10	y = 13.373x − 25.880	0.9996	1.2	2.5
Procyanidin C1	1.0–50	y = 12.580x − 38.568	0.9993	0.2	2.2
(-)-Epicatechin	1.0–50	y = 15.678x − 1.228	0.9997	0.4	1.7
Procyanidin A2	1.0–50	y = 15.207x − 2.578	0.9986	0.5	1.9
Gallic acid	0.5–100	y = 74.565x − 203.271	0.9997	1.6	1.9
Protocatechuic acid	0.2–20	y = 83.103x + 14.884	0.9998	0.3	0.9
5-O-Caffeoylquinic acid	0.5–100	y = 76.834x − 258.863	0.9994	2.9	3.1
Caffeic acid	0.1–50	y = 135.456x + 8.922	0.9998	0.5	0.6
p-Coumaric acid	0.1–10	y = 188.341x − 225.984	0.9989	0.03	0.1
Ferulic acid	0.1–10	y = 136.140x + 11.868	0.9999	0.1	0.2
Phloretin-2-O-β-glucoside	0.2–100	y = 55.217x − 34.456	0.9999	0.8	2.0
Quercetin-3-O-galactoside	5.0–250	y = 76.747x + 113.346	0.9987	2.5	3.7
Quercetin-3-O-glucoside	5.0–100	y = 67.725x − 92.729	0.9998	0.8	2.4
Quercetin-3-O-rutinoside	5.0–50	y = 51.333x − 21.087	0.9997	0.9	2.7
Quercetin-3-O-xyloside	0.5–100	y = 57.714x − 2.109	0.9942	0.8	2.4
Quercetin-3-O-arabinoside	5.0–200	y = 67.899x − 144.780	0.9993	2.7	4.2
Quercetin-3-O-rhamnoside	5.0–250	y = 76.344x − 300.286	0.9997	3.4	4.7
Quercetin	5.0–100	y = 77.325x − 305.333	0.9980	5.8	7.1

* The linearity was given over the entire 7-point calibration curve. c: concentration; LOD: limit of detection; LOQ: limit of quantification.
